# Integrated metagenomics and metabolomics analysis reveals changes in the microbiome and metabolites in the rhizosphere soil of *Fritillaria unibracteata*

**DOI:** 10.3389/fpls.2023.1223720

**Published:** 2023-08-04

**Authors:** Chengcheng Liu, Jingsheng Yu, Jizhe Ying, Kai Zhang, Zhigang Hu, Zhixiang Liu, Shilin Chen

**Affiliations:** ^1^ College of Pharmacy, Hubei University of Chinese Medicine, Wuhan, China; ^2^ Institute of Herbgenomics, Chengdu University of Traditional Chinese Medicine, Chengdu, China; ^3^ Institute of Chinese Materia Medica, China Academy of Chinese Medical Sciences, Beijing, China

**Keywords:** *Fritillaria unibracteata*, rhizosphere soil, metagenomics, cooccurrence analysis, metabolomics

## Abstract

*Fritillaria unibracteata* (FU) is a renowned herb in China that requires strict growth conditions in its cultivation process. During this process, the soil microorganisms and their metabolites may directly affect the growth and development of FU, for example, the pathogen infection and sipeimine production. However, few systematic studies have reported the changes in the microbiome and metabolites during FU cultivation thus far. In this work, we simultaneously used metagenomics and metabolomics technology to monitor the changes in microbial communities and metabolites in the rhizosphere of FU during its cultivation for one, two, and three years. Moreover, the interaction between microorganisms and metabolites was investigated by co-occurrence network analysis. The results showed that the microbial composition between the three cultivation-year groups was significantly different (2020-2022). The dominant genera changed from *Pseudomonas* and *Botrytis* in CC1 to *Mycolicibacterium* and *Pseudogymnoascus* in CC3. The relative abundances of beneficial microorganisms decreased, while the relative abundances of harmful microorganisms showed an increasing trend. The metabolomics results showed that significant changes of the of metabolite composition were observed in the rhizosphere soil, and the relative abundances of some beneficial metabolites showed a decreasing trend. In this study, we discussed the changes in the microbiome and metabolites during the three-year cultivation of FU and revealed the relationship between microorganisms and metabolites. This work provides a reference for the efficient and sustainable cultivation of FU.

## 1 Introduction

*Fritillaria* is a famous medicinal genus of Liliaceae, which includes more than 150 species (Protopopova et al., 2023). Fritillariae Cirrhosae Bulbus, the dried bulb of some *Fritillaria* plants, including *Fritillaria unibracteata* (FU), has been listed as a herbal medicine in the Chinese Pharmacopoeia (2020). It shows satisfactory therapeutic effects for the treatment of cough, bronchitis, and pneumonia and has a potential role in COVID-19 treatment (Niu et al., 2021). As one of the most important medicinal plant resources of Fritillaria Cirrhosae Bulbus (Chuan Beimu in Chinese), the quality of FU has received wide attention. The growth of FU requires multiple environmental factors, including altitude, temperature, and humidity, which causes its low yield. The harsh growth environment of FU forces growers to engage in continuous cropping. However, the pests and diseases have seriously affected the production and quality of FU (He et al., 2022). After two years of cultivation, Yan et al. (2022) observed that the production of *F. thunbergii* was reduced by more than half. He et al. (2022) also indicated that the quality of *F. taipaiensis* after three years of cultivation was affected by various factors, including the accumulation of diseases, growth damage, and quality degradation. Although various scientists have proposed distinct opinions, a conclusion has been accepted regarding which microorganisms and metabolites have remarkable effects on the quality of *Fritillaria* during its cultivation.

Microorganisms are widely distributed on Earth, and make great contribution to global biomass (Banerjee and van der Heijden, 2022). As the material support for terrestrial ecosystems, soil exhibits the most microbial community diversity and complexity (Dubey et al., 2019). Rhizosphere serves as the direct interface for energy and material exchange between plant and soil, and is also the primary habitat for microorganisms (Bardgett and van der Putten, 2014). Soil microorganisms colonized plant roots through mutualism or symbiosis to form complex rhizosphere microbial communities, thus influencing plant nutrient uptake and health (Philippot et al., 2013). The rhizosphere microbiome, also known as the “second genome” of plants, has a series of beneficial functions during plant growth and development, including nutrient acquisition, pathogen resistance, and stress tolerance (Sharma et al., 2020). It has been reported that many medicinal plants could recruit specific microorganisms through root exudates to resist biotic or abiotic stress as well as assisting the production of secondary metabolites, such as *Salvia milliorrhiza*, *Cannabis sativa*, *Panax notaginseng* (Yue et al., 2019; Li et al., 2020a; Vujanovic et al., 2020). These studies demonstrated that rhizosphere microbiomes made great contributions to medicinal plants. In addition, the rhizosphere microbiome can also affect the chemical composition and secretion of roots through systematic signaling mechanisms, thereby the metabolic composition of the rhizosphere soil of plants (Korenblum et al., 2020). The soil metabolites were mainly consisted of plant root secretions and small molecules released by soil microorganisms, such as sugars, amino acids, organic acids, phenolic compounds and other secondary metabolites (Cheng et al., 2022). For example, continuous cropping, fertilizer, and pesticide application significantly altered the metabolic composition of plant rhizosphere soil soils (Ge et al., 2022; He et al., 2022; Xu et al., 2023). Therefore, it is essential and important to reveal the changes in rhizosphere soil microorganisms and metabolites during plant growth and development for guiding plant production.

In this work, we used metagenomics and metabolomics technology through the Illumina MiSeq platform combined with the LC−MS/MS detection method to monitor the changes in microbial community structure and metabolites in the rhizosphere soil of FU during its three-year cultivation process. Furthermore, the differences in soil microbial communities and metabolites between three cultivation-year groups were compared. The interaction between microorganisms and metabolites was analyzed. This study revealed the rhizosphere soil microbial structure and metabolites of FU for the first time, providing a reference for standard settings in its practical cultivation.

## 2 Materials and methods

### 2.1 Location of the experiment and collection of soil samples from the rhizosphere

The rhizosphere soil samples of FU were collected from the Lv Lin Chuan BeiMu cultivation base (28°55′ N, 102°10′ E) in Mianning County, Sichuan Province, China. The cultivation area has a subtropical climate with an altitude of up to 2800 meters (m), an annual rainfall of 1095 millimeters (mm), an annual mean air temperature of 13.8°C and an annual evaporation of 1857 mm. The soil in the experimental area is dark brown loam. The 1000-seed weight of FU was approximately 1.2 grams (g), and the seeds were sown in March 2020, 2021 and 2022. The land was in a natural fallow state before sowing, and the FU was not transplanted after sowing. After mixing 5 g of seeds with 5 kilograms of humus, the seeds were sown per square meter in the test field and finally covered with approximately 1.5 centimeters (cm) of humus. Throughout the entire growth process of FU, no fertilizers or insecticides were used, and all plots were managed in the same way. The rhizosphere soil samples of FU for three consecutive years (years one, two, and three) were sampled on October 18 and 2022 and numbered CC1, CC2 and CC3, respectively (**Table 1**). Each treatment consisted of three replicates. Each replicate contained 10 samples and sampling was performed using the “Z” pattern. The specific sampling method was as follows: approximately 2-3 cm of surface soil was removed with a sampling shovel to expose the *Fritillaria* bulb, and the rhizosphere soil within 0.2 cm of the bulb was carefully scooped, filtered through a 100-mesh screen, and placed in a Ziplock bag. The samples were then shipped to the laboratory in ice boxes, immersed in liquid nitrogen for at least 5 minutes, and stored at -80°C.

**Table 1:** Detailed information for *Fritillaria unibracteata* rhizosphere soil sample.

Sampling information								
Host Latin name	Cultivation year	Group	Sample id	Planting date	Sampling date	Elevation	Sampling temperature	SAMN number
*Fritillaria unibracteata*	one	CC1	CC1a	Mar-2020	Oct. 18, 2022	2800 meters	4°C	SAMN34413994
*Fritillaria unibracteata*	one		CC1b	Mar-2020	Oct. 18, 2022	2800 meters	4°C	SAMN34413995
*Fritillaria unibracteata*	one		CC1c	Mar-2020	Oct. 18, 2022	2800 meters	4°C	SAMN34413996
*Fritillaria unibracteata*	two	CC2	CC2a	Mar-2021	Oct. 18, 2022	2800 meters	4°C	SAMN34413997
*Fritillaria unibracteata*	two		CC2b	Mar-2021	Oct. 18, 2022	2800 meters	4°C	SAMN34413998
*Fritillaria unibracteata*	two		CC2c	Mar-2021	Oct. 18, 2022	2800 meters	4°C	SAMN34413999
*Fritillaria unibracteata*	three	CC3	CC3a	Mar-2022	Oct. 18, 2022	2800 meters	4°C	SAMN34414000
*Fritillaria unibracteata*	three		CC3b	Mar-2022	Oct. 18, 2022	2800 meters	4°C	SAMN34414001
*Fritillaria unibracteata*	three		CC3c	Mar-2022	Oct. 18, 2022	2800 meters	4°C	SAMN34414002

### 2.2 Determination of the physicochemical properties of the rhizosphere soil

The measurement of pH, organic matter (OM), dissolved organic carbon (DOC), cation exchange capacity (CEC), total nitrogen (TN), nitrate nitrogen (NO3-N), ammonium nitrogen (NH4-N), total phosphorus (TP), total potassium (TK), available phosphorus (AP), and available potassium (AK) in rhizosphere soils was performed as described by (Song et al., 2020).

### 2.3 Soil metabolome analysis

One gram of soil sample was accurately weighed and placed in a 2 ml centrifuge tube, and then 1 milliliter of aqueous methanol solution (80%, V/V, Macklin) was accurately pipetted. Then, the centrifuge tube containing the sample was placed in an ice-water mixture for 10 minutes and vortexed for 5 minutes to completely dissolve the soil sample. After dissolving, it was placed in a high-speed centrifuge at 12000 rpm, centrifuged at 4°C for 15 minutes. The supernatant was carefully aspirated and centrifuged again for 30 minutes. The supernatant was gently aspirated again, freeze dried and stored at -80°C for subsequent use. Prior to injection, the lyophilized samples were resuspended in 200 microliters of methanol, and the instruments used for LC−MS/MS analysis were a Vanquish Neo UHPLC system (Thermo Scientific) and Q Active HF-X (Thermo Scientific). The chromatographic column was a Hypseril Gold (100 × 2.1 mm, 1.9 µm, Thermo Fisher) column, and the chromatographic conditions were as follows: flow rate of 0.2 ml per minute and linear gradient elution for 12 minutes. The eluent consisted of methanol (B), an aqueous 0.1% formic acid solution (positive ion mode) and ammonium acetate at pH 9.0.

### 2.4 DNA extraction, library construction, and metagenomic sequencing

The extraction of DNA from 0.5 g of soil was performed using the DP336-02 TIANamp Soil DNA Kit (Omega Biotek, Inc., Norcross, GA, USA) according to the manufacturer’s instructions. The concentration of soil DNA was determined using a NanoDrop 2000-UV spectrophotometer (Thermo Scientific, Waltham, MA, USA), and the quality of the DNA was examined using 1% agarose gels. A library was prepared using the NEB Next^®^ Ultra™ DNA Library Prep Kit for Illumina (NEB, USA). Qualified DNA samples were randomly fragmented into 350 bp fragments using a Covaris (Covaris S2 System, Massachusetts,USA) ultrasonic fragmentation instrument. A complete library was constructed by terminus repair, polyA tailing, sequence linking, purification, PCR amplification and other steps. Finally, the AMPure XP system was used to purify the PCR products, an Agilent 2100 was used to determine the insert size of the library, and real-time PCR was used for quantitative analysis of the library concentration. The indexed coding samples were clustered on the cBot Cluster Generation System using the Illumina PE Cluster Kit (Illumina, USA) according to the manufacturer’s instructions. After clustering, the DNA library was sequenced on the Illumina NoVaseq 6000 platform, and 150 bp paired-end reads were generated.

### 2.5 Statistical analysis

This experiment investigated the metabolome of the sample by LC−MS/MS. The specific procedure was as follows: (1) The original data obtained were converted to mzXML format (xcms input file format) using Proteowizard software (v3.0.8789). (2) The R (v3.1.3) XCMS package was used to perform peak identification, peak filtering, and peak alignment. (3) The mass to charge ratio (m/z), retention time, and intensity data matrix were obtained. Finally, the precursor molecules were obtained in positive and negative ion modes, and the data were exported for further analysis.

Macrogenomic analysis was performed based on sequencing reads. The original sequencing data were preprocessed using Kneaddata software to ensure data availability. The number of sequences of the species present in the sample were determined using Kraken2 and a custom microbial nucleic acid database. Bracken was used to predict the actual relative abundance of species in the sample. The quality control and host-removed sequences were compared with the DIAMOND-based protein database UniRef using HUMAnN2 software. Based on the correspondence between UniRef ID and various databases, annotation information and relative abundance tables were obtained for each functional database.

SPSS 20.0 (IBM) was used for analysis of variance (ANOVA), and R version 4.2.2 was used for data visualization. Microbial alpha diversity was achieved using the vegan and picante packages, co-correlation analysis between microorganisms and metabolites was performed using the Igraph package and psych package. Procrustes analysis and principal coordinate analysis (PCoA) analysis were performed using the vegan package, and cluster heatmap analysis was performed using the ComplexHeatmap package. Linear discriminant analysis effect size (LEfSe) analysis was performed at https://www.bioincloud.tech. Microbial function prediction was conducted using FAPROTAX 1.2.6 software.

## 3 Result

### 3.1 Physicochemical properties of rhizosphere soil

The physicochemical properties of the rhizosphere soil during the three-year cultivation period of FU are shown in **Supplementary Table 1**. There was no significant difference in the contents of TK, AK, NH_4_-N and DOC among the CC1, CC2 and CC3 groups. During the three years of cultivation, the pH level of the FU rhizosphere soil increased in CC2 and then decreased in CC3. Similar change trend was also observed for TN and TP, but the CC3 group was still significantly higher than the CC1 group. Notably, the AP content showed a significant increasing trend from the CC1 to CC3 groups.

### 3.2 Microbial diversity in various CC samples

**Figure 1:**
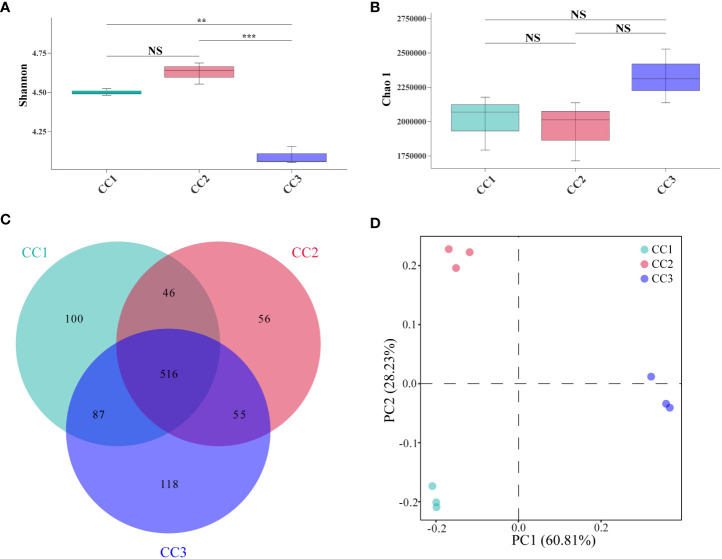
Shannon index **(A)** and Chao 1 index **(B)** of bacterial communities. Venn diagram of genera in different groups **(C)**. PCoA analysis of bacterial communities in three groups **(D)**. **Indicating very significant differences between groups, p<0.01; ***Indicating extreme significant differences between groups, p<0.001; NS indicates no significant difference between groups.

**Figure 2:**
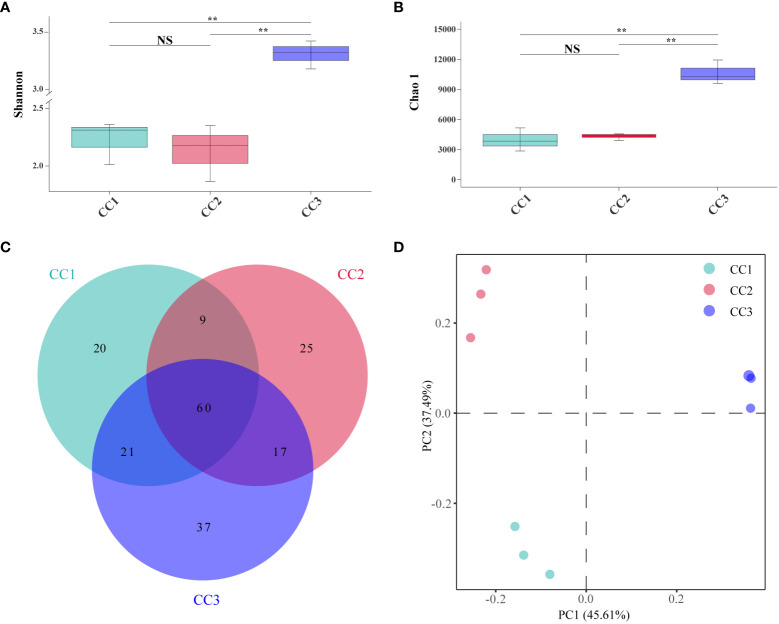
Shannon index **(A)** and Chao 1 index **(B)** of fungal communities. Venn diagram of genera in different groups **(C)**. PCoA analysis of fungal communities in three groups **(D)**. **Indicating very significant differences between groups, p<0.01; NS indicates no significant difference between groups.

A total of 215,662,114 raw reads were derived from 9 samples after Illumina sequencing in this study. A total of 199,835,955 clean reads were obtained after quality control, and there was sufficient sequencing depth to reflect the microbiome structure in each sample (**Supplementary Table 2**). The Shannon index of the bacterial community did not change significantly between the CC1 and CC2 groups but decreased significantly (4.63-4.09) in the CC3 group. No significant differences were observed in the Chao1 index of the bacterial community among the CC1, CC2 and CC3 groups (**Figures 1A, B**; **Supplementary Table 3**). The Shannon (2.13-3.14) and Chao1 (4313-10607) indices of the fungal community increased significantly in the CC3 group (**Figures 2A, B**; **Supplementary Table 4**). These results showed that the α-diversity of the bacterial community decreased significantly during FU cultivation, and the fungal community diversity and richness increased significantly.

### 3.3 Composition of the bacterial community in various CC samples

**Figure 3:**
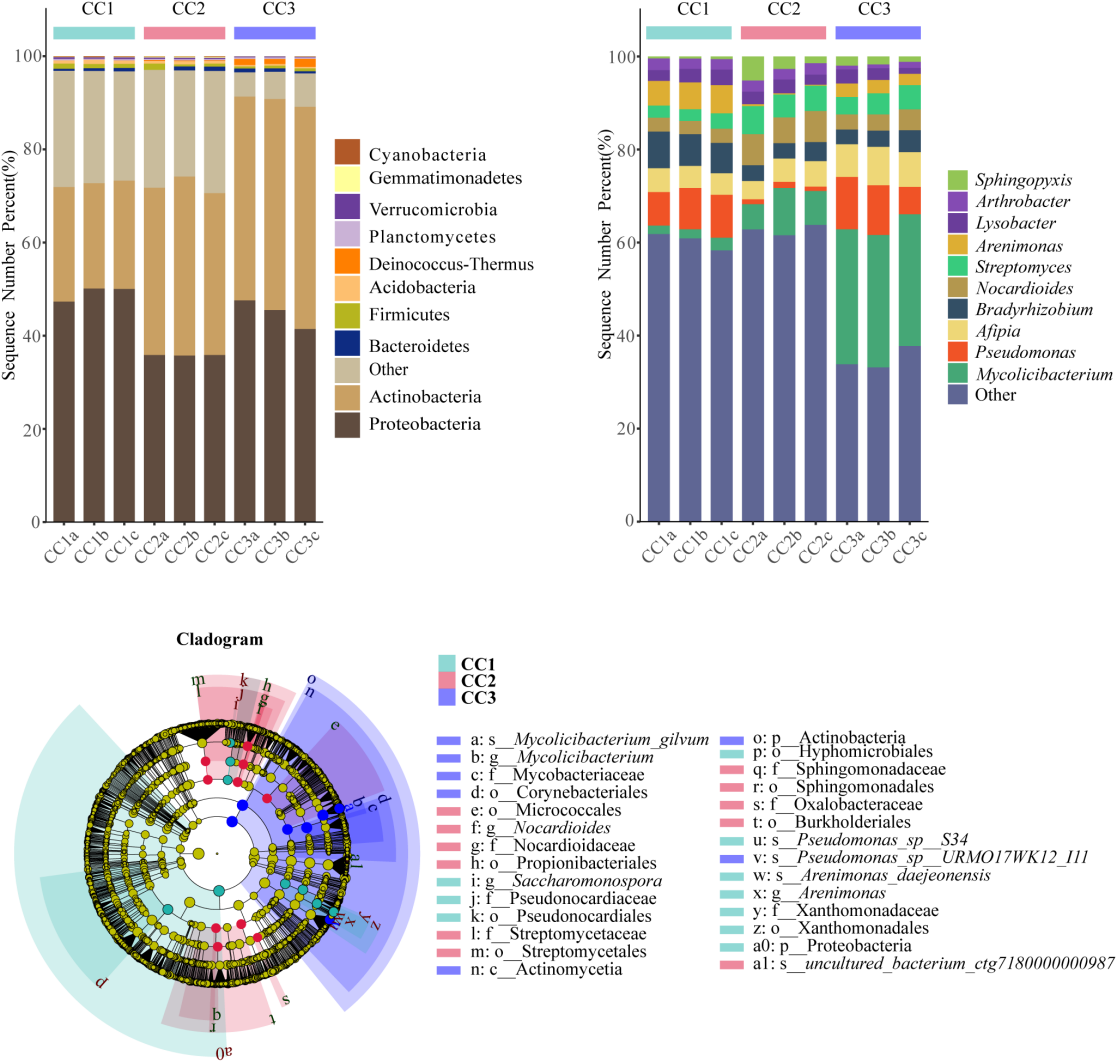
The composition of rhizosphere soil bacterial phylum **(A)** and genus **(B)** levels during FU cultivation (only Top10 is shown). Cladogram from LEfSe analysis showed changes in the abundance of bacterial at different taxonomic levels **(C)**.

From the phylum to the species level, metagenomic sequencing identified 40, 63, 143, 318, 977, and 3803 taxa, respectively. A total of 749, 673, and 776 genera were detected in CC1, CC2, and CC3, respectively (**Figure 1C**). Proteobacteria had the highest relative abundance among all phyla, followed by Actinobacteria, Bacteroidetes, Firmicutes, and Acidobacteria (**Figure 3A**). During cultivation, the relative abundance of Actinobacteria increased with cultivation time, while the relative abundance of Acidobacteria decreased. Interestingly, the relative abundance of Proteobacteria and Firmicutes showed a trend of first increasing and then decreasing during the cultivation process, while the relative abundance of Bacteroidetes showed an opposite trend (decreasing first and then increasing). Actinomycetia, Corynebacteriales, and Mycobacteriaceae were dominant at the class, order, and family levels. We further analyzed the bacterial community at the genus level to better understand the changes in bacterial abundance and composition during cultivation. *Mycolicibacterium*, *Pseudomonas*, *Afipia*, *Bradyrhizobium*, and *Nocardioides* were more abundant than other genera (**Figure 3B**). The cluster heatmap (**Supplementary Figure 1**) showed that the relative abundances of some bacterial genera, such as *Afipia*, *Mycolicibacterium*, *Deinococcus*, *Rhodanobacter* and *Nitrosospira*, were higher in the CC3 group than those in the other groups. The relative abundances of *Micromonospora*, *Sphingopyxis*, *Massilia*, *Nocardioides*, *Sphingomonas* and *Ralstonia* were higher in the CC2 group, and the relative abundances of *Methylocaldum*, *Thermobifida*, *Bradyrhizobium Saccharomonospora* and *Thermomonospora* were higher in the CC1 group. The beta diversity of the bacterial community in the CC1, CC2 and CC3 groups was analyzed by PCoA (**Figure 1D**). PC1 explained 60.81% of the variation, while PC2 explained 28.23%. The PCoA results revealed that the three groups could be distinguished, indicating that cultivation time affected the structure of the microbial community in the rhizosphere soil of FU. This result was also supported by Bray−Curtis clustering analysis (**Supplementary Figure 2**). Based on LEfSe analysis, the three groups showed significantly differences in the relative abundances of bacterial species (**Figure 3C**; **Supplementary Figure 3**). Twenty-eight biomarkers were enriched across the samples with LDA scores >4. Among these biomarkers, there were seven taxa in CC3, with *Mycolicibacterium gilvum*, a rarely reported bacterium in cultivation soil, being the largest contributor. There were 11 taxa in CC2, and the major contributors were Propionibacteriales, Burkholderiales, Nocardioidaceae, Sphingomonadaceae, and *Nocardioides*. There were ten taxa in CC1, and the major contributors were Proteobacteria and *Arenimonas daejeonensis*.

### 3.4 Composition of the fungal community in various CC samples

**Figure 4:**
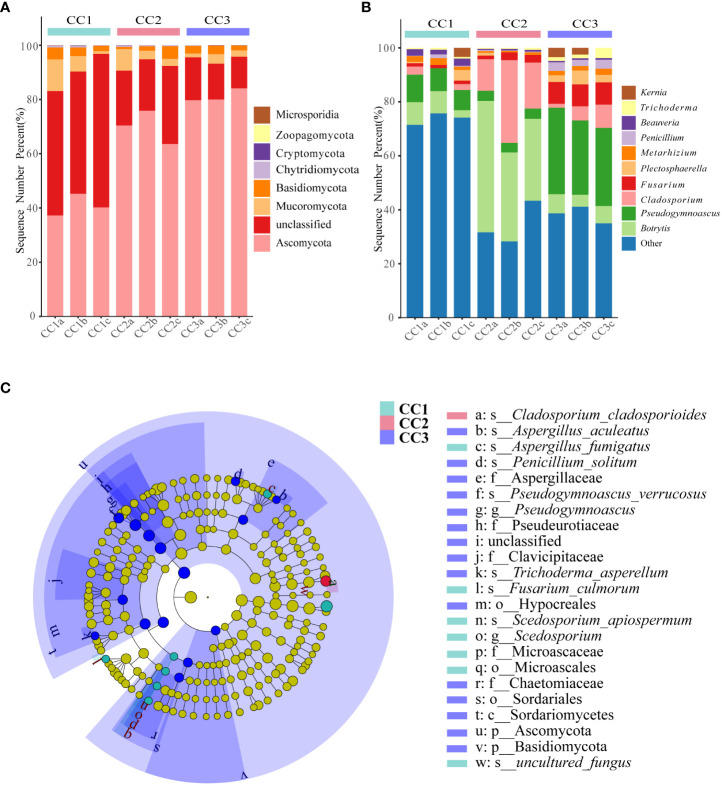
The composition of rhizosphere soil fungal phylum **(A)** and genus **(B)** levels during FU cultivation (only Top10 is shown). Cladogram from LEfSe analysis showed changes in the abundance of fungal at different taxonomic levels **(C)**.

From the phylum to the species level, metagenomic sequencing identified 7, 24, 60, 119, 188, and 345 taxa. There were 110, 111 and 135 genera that were identified in CC1, CC2 and CC3, respectively (**Figure 2C**). This result indicated that cultivation increased the number of fungal genera in the rhizosphere soil of FU. The relative abundances of Ascomycota (64.02%), Mucoromycota (4.37%), and Basidiomycota (2.72%) were higher than those of other phyla. The relative abundance of Ascomycota significantly increased (13.6%-27.1%) with cultivation time, while the relative abundance of Mucoromycota decreased (**Figure 4A**). At the genus level, *Botrytis*, *Pseudogymnoascus*, *Cladosporium* and *Fusarium* were abundantly present in the FU samples (>2%) (**Figure 4B**; **Supplementary Table 5**). The relative abundances of these genera were higher in CC2 or CC3 than in CC1. Furthermore, the relative abundance of *Penicillium* increased significantly (2.1% - 9.9%) in CC3. The relative abundances of *Beauveria* and *Aspergillus* were higher in CC1 than in the other groups (**Supplementary Figure 4**). PCoA was performed based on soil fungal communities (**Figure 2D**). PC1 explained 45.61% of the variance, and PC2 explained 37.49% of the variance. Similar to the results of bacterial PCoA, the three groups were distinct (**Supplementary Figure 2**). The LEfSe analysis results showed that CC1 had the seven out of the 23 biomarkers with LDA>4, with two ubiquitous fungi, *Fusarium culmorum* and *Aspergillus fumigatus*. In addition, the beneficial fungal genus *Scedosporium* was also a biomarker of CC1. In CC2, only *Cladosporium cladosporioides* was a biomarker. There were 15 taxa in CC3, and the major contributors were Ascomycota and *Pseudogymnoascu* (**Figure 4C**; **Supplementary Figure 3**).

### 3.5 Potential functional pathways in FU rhizospheric soil microbes

**Figure 5:**
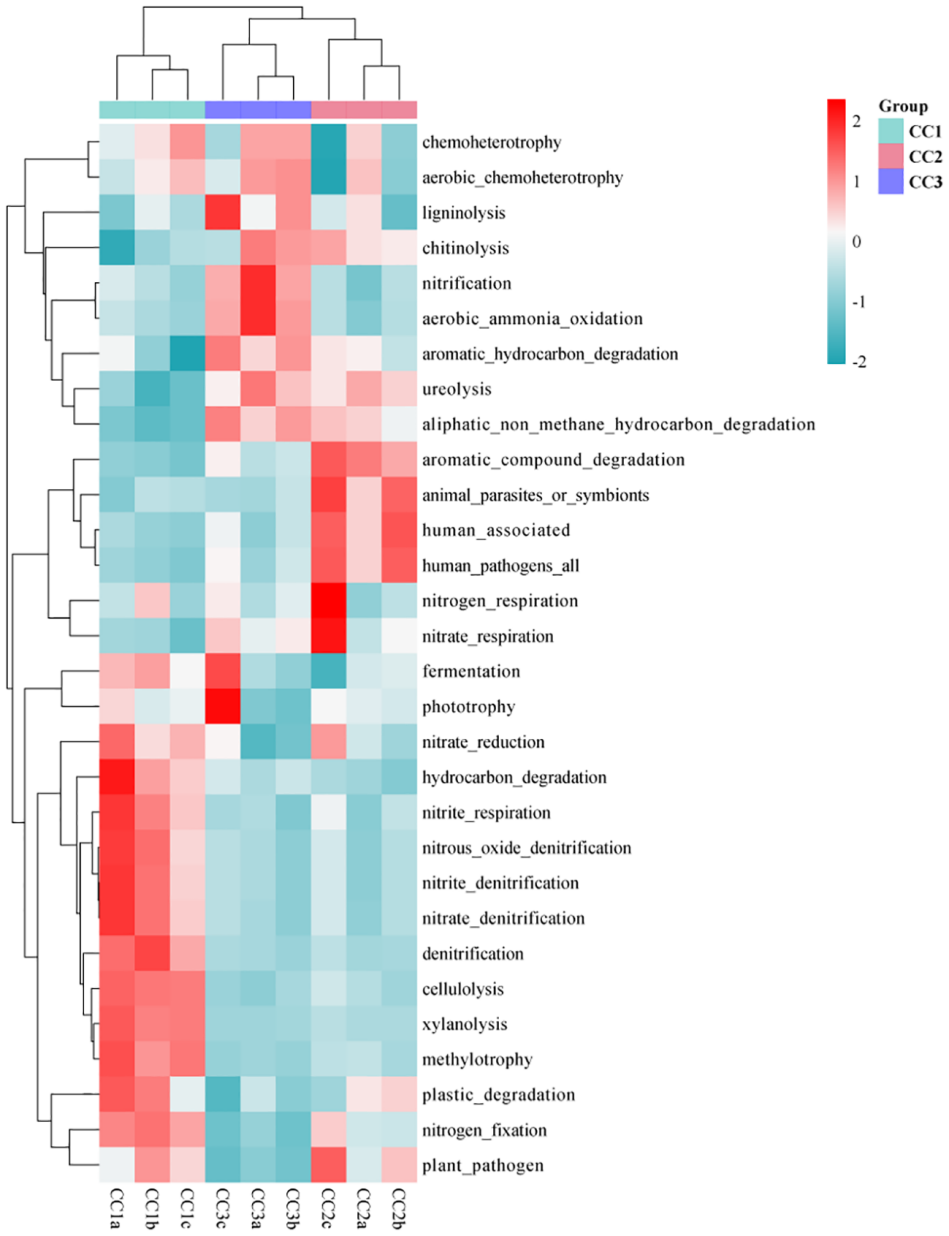
Functional heat map of microbial community in FAPROTAX database of three groups.

As illustrated by the Venn diagram (**Supplementary Figure 5**), a total of 355 level 3 pathways were annotated in the KEGG database for all samples, of which 325, 341, and 325 pathways were detected in CC1, CC2, and CC3, respectively. Among them, there were 4, 24, and 3 unique pathways, respectively. The annotation results show that the dominant categories were Ribosome, Valine, leucine and isoleucine biosynthesis, Synthesis and degradation of ketone bodies, Lipoic acid metabolism, and Cell cycle - Caulobacter, representing 3.21%, 2.97%, 2.70%, 1.96%, and 1.91% of the total, respectively. Furthermore, among all categories, the relative abundances of genes related to the Citrate cycle (TCA cycle), Carbon fixation in photosynthetic organisms, Valine, leucine and isoleucine degradation increased during the three cultivation stages. However, the relative abundances of genes associated with Atrazine degradation, Steroid hormone biosynthesis, and Polycyclic aromatic hydrocarbon degradation decreased with the cultivation years (**Supplementary Table 6**). In the FAPROTAX database of microbial ecological function predictions, the annotated OTUs were assigned to 90 predicted functional groups (**Supplementary Table 7**). Nevertheless, in the ANOVA test, only 30 groups showed significant differences between the three compartments (*P* < 0.05). Therefore, the data were plotted as a functional heatmap (**Figure 5**). The results showed that the CC1 soil was enriched with the denitrification of nitrogen, methylotrophy, methylotrophy, and hydrocarbon degradation. CC2 was enriched with nitrogen respiration, nitrate respiration, and aromatic compound degradation. CC3 was enriched with nitrification, aerobic ammonia oxidation, and aromatic hydrocarbon degradation.

### 3.6 Analysis of nontargeted soil metabolites

**Figure 6:**
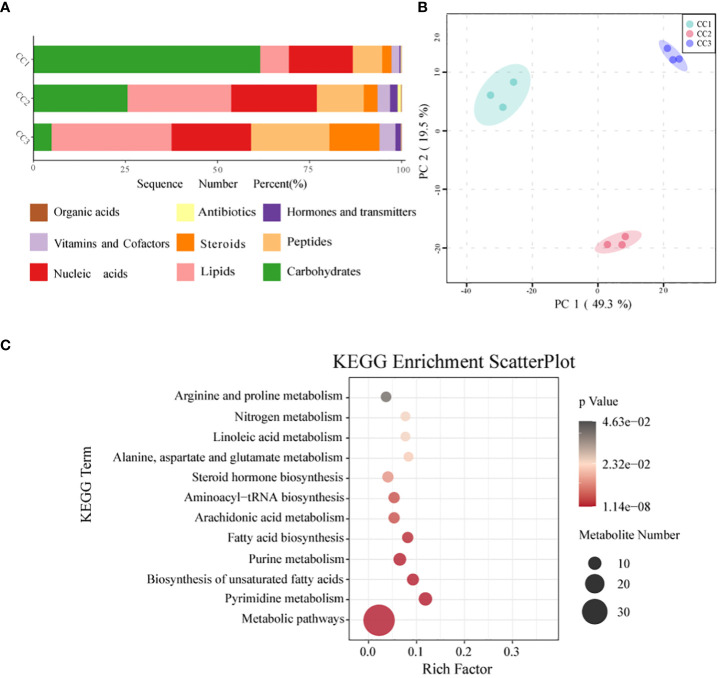
Metabonomics analysis of three groups. **(A)** Level of functional metabolite content **(B)** PCA analysis of metabolites in three groups. **(C)** KEGG pathway enrichment analysis of soil metabolic profiles. *P* <.05 indicated that the pathway was significantly enriched. The size of the bubbles and the “Numbers” legend in this image represents the amount of metabolites that are. Enriched. The size of the bubbles and the “numbers” legend in this image represents the amount of metabolites that are concentrated in this pathway.

In all rhizosphere soil samples, a total of 1087 compounds were detected. After annotation of br08001 in the KEGG database, 103 compounds with biological functions were obtained, among which the relative content of carbohydrates was the highest (44.16%), followed by nucleic acids (19.06%) and lipids (16.19%) (**Figure 6A**; **Supplementary Table 8**). With increasing years of cultivation, the relative content of carbohydrates in the rhizosphere soil decreased sharply, but the relative content of lipids, peptides, and steroids increased. According to the principal component analysis (**Figure 6B**), the CC1, CC2 and CC3 groups showed significant differences in the metabolite composition of the rhizosphere soil. PC1 accounted for 49.3% of the total variation, while PC2 accounted for 19.5%. **Supplementary Figure 6** and **Supplementary Table 9** list the top 20 compounds with the highest relative abundance that had biological functions. Although there was only one type of carbohydrate (sucrose), its relative abundance was indeed the highest at 29.59% and it was also one of the compounds with the most significant differences (**Supplementary Table 10**). This was followed by palmitic acid (19.43%), adenosine (9.97%), L-phenylalanine (8.68%) and prostaglandin J2 (2.09%). The metabolites with biological functions with the most significant differences among the three groups were screened to determine the metabolites that differed significantly among the groups, with VIP > 1 as the criterion. The top 10 metabolites with significant differences were prostaglandin E1, sucrose, cytidine, docosahexaenoic acid, palmitoleic acid, myristic acid, serotonin, L-hydroxyproline, oleic acid, and 2-deoxyuridine. The relative contents of sucrose, palmitoleic acid, myristic acid and oleic acid decreased with increasing cultivation time. The relative abundances of serotonin, L-hydroxyproline and 2-deoxyuridine increased with cultivation time. To elucidate the specific changes in soil metabolic processes, pathway enrichment analysis was performed. The metabolic pathway was the most significantly altered pathway in the rhizosphere soil, as shown in **Figure 6C**. Pyrimidine metabolism, unsaturated fatty acid biosynthesis, purine metabolism, fatty acid biosynthesis, and nitrogen metabolism were also significantly enriched.

### 3.7 Correlations between soil bacteria, fungi, and metabolism

**Figure 7:**
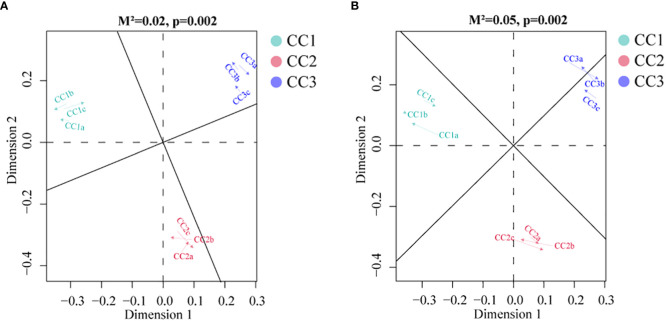
Procrustes analyses of metabolic profiles at bacterial **(A)** and fungal **(B)** taxa.

**Figure 8:**
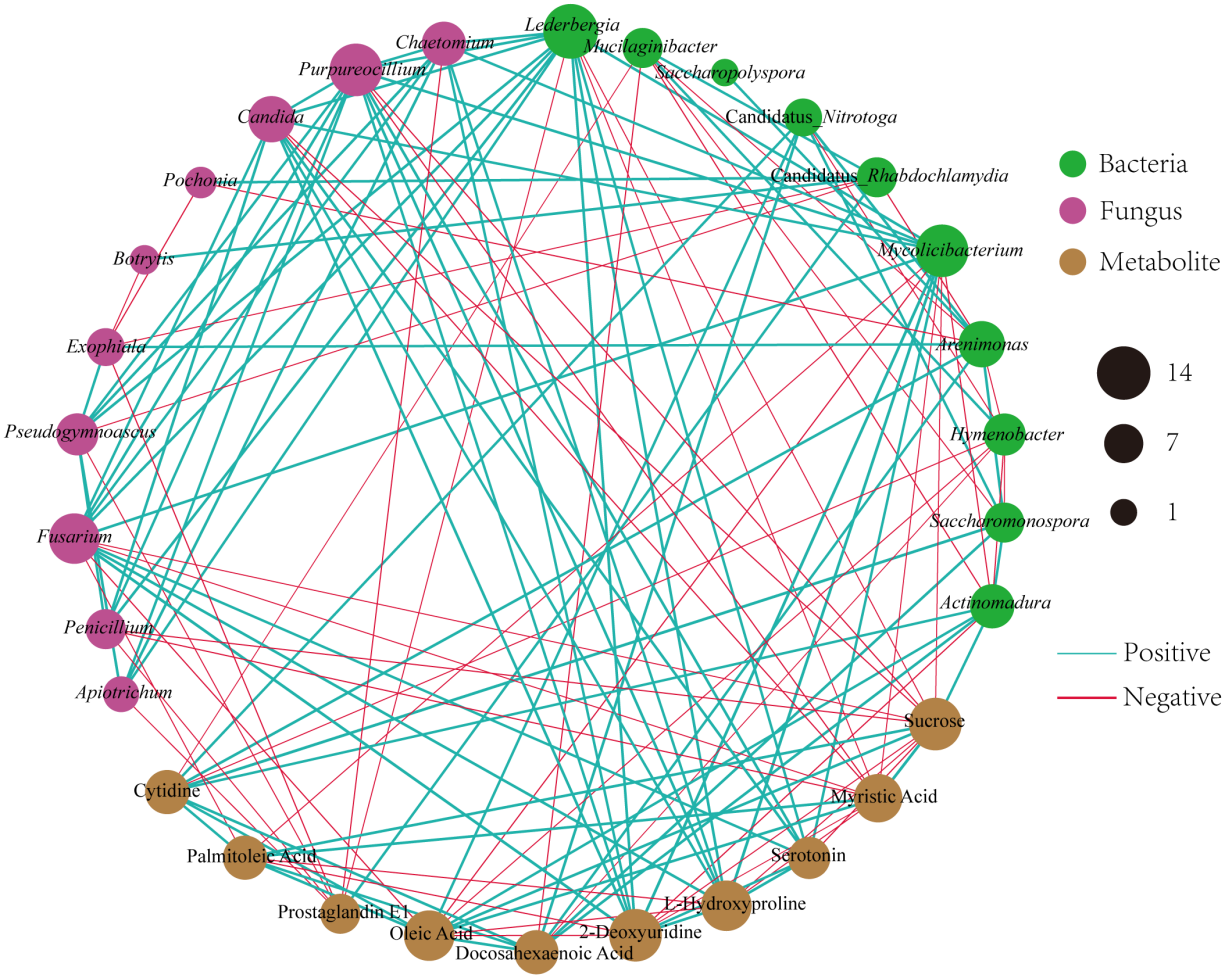
Different microbial genera and metabolites of Co-occurrence network. The red line shows a negative correlation, and the green line shows a positive correlation. The thicker the line, the stronger the correlation.

To investigate the relationships among the bacterial groups, the fungal groups and the soil metabolites, the Bray−Curtis distance was used to perform the Procrustes analysis. The results showed that the correlation between bacterial groups and soil metabolites (**Figure 7A**, *M^2 ^= *0.02, *P*=0.002) was higher than that between fungal groups and soil metabolites (**Figure 7B**, *M^2 ^= *0.05, *P*=0.002). An interaction network (**Figure 8**) was constructed to further elucidate the relationships between differentially abundant metabolites and differentially abundant microbes in the rhizosphere. In general, the networks of metabolites or microorganisms were more positively correlated than negatively correlated. However, there was a more negative correlation between differential metabolites and differential microorganisms. For example, sucrose, palmitoleic acid and oleic acid have differential microbes. The varying sizes of soil microbial nodes indicated that these different microorganisms contributed differently to metabolism. Among the differential metabolites, L-hydroxyproline was positively correlated with the microbial genera with which it was significantly associated. The opposite was observed for myristic acid. Cytidine and docosahexaenoic acid were significantly correlated with only differentially abundant bacteria, not with differentially abundant fungi. Among the differentially abundant microbial genera, the greatest number of differentially abundant metabolites were significantly associated with the differentially abundant bacterial genus *Mycolicibacterium*, while *Saccharopolyspora* was not significantly associated with any differentially abundant metabolite. The greatest number of differentially abundant metabolites was significantly associated with the differentially abundant fungal genus *Fusarium*, while *Pochonia* and *Botrytis* were not significantly associated with any differential metabolites. Moreover, the correlation heat map analysis (**Supplementary Figure 7**) results showed that *Botrytis* had significant positive correlations (*P*<0.05) with Candidatus_*Rhabdochlamydia*, *Hymenobacter* and *Mucilaginibacter*, while it showed highly significant negative correlations (*P*<0.01) with *Arenimonas* and *Saccharomonospora*. *Lederbergia* and *Mycolicibacterium* showed positive correlations with almost all fungal genera. In contrast, *Saccharopolyspora* showed negative correlations with all fungal genera. We conducted a correlation heat map analysis between the microbial genus and the top 20 differential metabolites, in order to identify metabolites specifically related to microorganisms (**Supplementary Figure 8**). Among the 30 bacterial genera with the highest degree of significant correlation with differential metabolites, all genera showed significant correlations with Prostaglandin J2, L-Glutamic acid, and Uridine, with a positive correlation exceeding a negative correlation. In addition, most bacterial counts are significantly correlated with 6-Deoxy-D-glucose, Sucrose, Adenine, and Guanine, with a positive correlation more than a negative correlation. For fungi, there is more negative correlation between fungi and metabolites than positive correlation. The metabolites most significantly associated with fungi are Prostaglandin J2 and 6-Deoxy-D-glucose, followed by L-Glutamic acid, Sucrose, and Uridine, which also have a significant correlation with the bacterial genus. From this, it can be seen that Prostaglandin J2, 6-Deoxy-D-glucose, L-Glutamic acid, Sucrose, and Uridine are specific metabolites highly associated with microorganisms during FU cultivation.

## 4 Discussion

### 4.1 Cultivation affects the structure of microbial communities and increases the relative abundance of pathogens

With the intensification of agricultural development, it is becoming increasingly common to grow the same crop continuously on the same cultivated land. Monocultures are considered likely to lead to pathogen accumulation and deterioration of soil conditions (Liu et al., 2018). Monocultures can also cause continuous cropping disorders, as observed for *Panax quinquefolius*, *Pinellia ternata*, and *Lilium lancifolium* (Dai et al., 2022; Li et al., 2022; Bi et al., 2023). The soil microbial communities and metabolites were considered non-ignorable factor that affected the plant growth and development in single cultivation practices based on the previous studies (Lu et al., 2022; Zhang et al., 2022). On the one hand, rhizosphere microorganisms have the potential to assist host plants in taking up nutrients and resisting biotic/abiotic stress (Liu et al., 2020; Choi et al., 2021; Zhang et al., 2021). On the other hand, some harmful rhizosphere microorganisms can also cause adverse effects on plants, such as reduced resistance and reduced yield (Mansfield et al., 2012; Ullrich et al., 2019). Therefore, rhizosphere microorganisms play an important role in the growth and development of host plants. In this study, we applied metagenomics and metabolomics technologies to evaluate the effect of cultivation time on rhizosphere microorganisms and soil metabolites. The results showed that the alpha diversity indices of the bacterial community decreased significantly after three years of cultivation, while the alpha diversity indices of the fungal community increased significantly. This was similar to the study on continuous cultivation of ginseng conducted by Dong et al. (2018). Changing the “fungal type” was obvious, which would lead to deteriorating the soil microecological environment (Li et al., 2017). Additionally, the microbial community composition in this work was similar to that in previous reports. Proteobacteria was the most common phylum among all bacteria. During the three years of cultivation, the changes in the relative abundance of Proteobacteria showed a decreasing trend followed by an increasing trend. It was reported that these bacteria showed the ability to decompose organic matter and promote plant growth (Chaudhry et al., 2012). In addition, Actinomycetes play an important role in agriculture because they can indirectly assist plants in defending against pathogens and pests (Borah et al., 2022). Dai et al. (2018) indicated that most Actinomycetes were saprophytes, and the abundance of Actinobacteria increased in soils with long periods of intense production. Therefore, the reason for the increased relative abundance of Actinomycetes in this study might have occurred response to the increase in pathogens. To combat the accumulation of soil-borne pathogens associated with continuous crop disturbance, studies have shown that more biocontrol bacteria are needed to ensure plant health (Siegel-Hertz et al., 2018; Tariq et al., 2020). *Pseudomonas* species are important biocontrol bacteria of special interest for their ability to control a wide range of soil-borne plant diseases (Biessy and Filion, 2021). Interestingly, in our results, the relative abundance of *Pseudomonas* showed a decreasing trend followed by an increasing trend. However, the relative abundance of another important biotrophic fungal genus, *Streptomyces*, increased significantly with increasing years of cultivation. The relative abundance of the harmful bacterial genus *Nocardioides* gradually increased during the cultivation of FU. We speculate that *Streptomyces* may have an interactive relationship with *Nocardoides*, and *Pseudomonas* may be involved in the prevention and control of *Nocardoides* infection in the later stage. In this work, we observed that *Mycolicibacterium gilvum*, a bacterium rarely reported in cultivation soil, had a relative abundance that increased dramatically in the continuous cropping process of *FU*. This bacterial species might have the potential for application as a biocontrol agent in FU cultivation in the future. Among fungi, the relative abundance of Ascomycetes, which are sap-sucking fungi, increased. They display the ability to decay and decompose litter and are a major source of toxins that cause plant diseases. *Botrytis* species are known to cause disease in more than a thousand species of plants (Veloso and van Kan, 2018). The relative abundance of *Botrytis* in the rhizosphere soil increased significantly during the second year of FU cultivation, so the gray mold rot that *Botrytis* can induce during the second year of planting (Veloso and van Kan, 2018) should be considered. The relative abundance of other pathogenic fungal genera, *Pseudogymnoascus* and *Plectosphaerella*, increased significantly in the third year of cultivation. The relative abundance of *Fusarium*, a typical pathogenic genus, increased significantly in medicinal plant soils (Zhang et al., 2020). In summary, the structure of the microbial community in the cultivation soil of FU shifted from the bacterial type to the fungal type, and the relative abundance of the pathogens in the soil increased. Our research results can provide a reference for the efficient detection and identification of pathogenic bacteria.

### 4.2 Cultivation affects the composition of metabolites in FU rhizosphere soil

It is crucial to clarify how rhizosphere metabolism regulates soil-microbe−plant interactions, which could provide insight into the feedback mechanisms of different plants necessary for plant health and improved crop yields (Bi et al., 2021; Korenblum et al., 2022). The results of PCoA showed that the metabolic composition of the rhizosphere soil changed significantly during the cultivation of FU. According to the VIP and p values of OPLS-DA, significantly differently abundant metabolites were screened out. Sucrose was the most depleted metabolite. The bulbs of FU were mainly composed of carbohydrates such as starch, and sucrose conversion affects the storage of starch. This implied that a large amount of sucrose in the soil was consumed in the third year of FU cultivation to meet the needs of FU growth. In addition, green manure application significantly increased the levels of beneficial metabolites (such as sucrose) in peanut soil (Xu et al., 2023). Therefore, some organic fertilizers, such as green manure, should be applied in a timely manner during the cultivation of FU based on our study. Additionally, the relative abundances of some root secretions such as sucrose and glucose, which were considered as the nutrients and energy substances for microorganism, significantly reduced during FU cultivation (**Figure 6**; **Supplementary Table 8**). Based on the previous studies, the low level of these secretions might affect the growth and development of microorganisms that used sugars as a carbon source, such as *Thermobifida*, *Beauveria* and *Aspergillus* (Chen et al., 2006; Gultom et al., 2014; Zhou et al., 2014). Similarly, our results demonstrated that the relative abundances of these fungi were related with the sucrose and glucose level (**Supplementary Figures 1**, **4**). In contrary, the relative abundances of two other important root secretions, nucleic and organic acids, increased during FU cultivation (**Figure 6**). Similar change trends were observed in the root secretions of Arabidopsis after pathogen infection (Yuan et al., 2018), suggesting that the risk of pathogen infection should be monitored during FU cultivation. As a result of the lack of phosphorus fertilizer, the abundance of organic acid abundance increased (Khorassani et al., 2011). In this study, the AP content in the rhizosphere soil continuously increased with FU cultivation, and correspondingly, the relative abundances of organic acid continued to decrease (**Supplementary Table 1**). The relative abundances of some microorganisms closely related to root exudates, such as *Arthrobacter*, *Bacillus*, *Azospirillum*, *Serratia*, and *Azotobacter*, exhibited a decreasing trend during FU cultivation. Previous studies have shown that they could assist plants to resist stress such as salt and alkali (Singh and Jha, 2016; Vives-Peris et al., 2020). This reminds us to provide a stable growth environment for FU in the later stage of cultivation. In recent years, much information has emerged about the antimicrobial potential of palmitoleic acid. Research has shown that palmitoleic acid derivatives exhibit high inhibitory activity against pathogens such as *Streptococcus mutans*, *Candida albicans*, *Fusobacterium nucleatum*, and *Porphyromonas gingivis* (Huang et al., 2010). Palmitoleic acid also inhibited spore germination of *Erysiphe polygoni*, a pathogen that causes powdery mildew (Wang et al., 2002). (Morgunov et al., 2017) reported that transgenic eggplants with high palmitoleic acid content also showed increased resistance to *Verticillium* wilt. According to our results, the relative abundance of palmitoleic acid decreased with the time of cultivation, so it is necessary to be vigilant for the diseases caused by these pathogens during the cultivation of FU. Furthermore, the relative abundances of other beneficial metabolites (myristic acid and oleic acid), which have been reported to have antibacterial and antifungal potential, also showed a decreasing trend (Liu et al., 2008; Kim et al., 2022). Serotonin is a potent antioxidant. In plants, phytoserotonin has been found to have many functions, including the regulation of plant growth and development, photosynthesis, reproduction, and responses to biotic and abiotic stresses (Raza et al., 2022). Thus, serotonin has been considered a biomarker for biotic or abiotic stress. The relative abundance of serotonin in the rhizosphere soil increased during FU cultivation, suggesting that FU might be affected by biotic or abiotic stress. Dar et al. (2016) reported that L-hydroxyproline plays an important role in regulating cell division, cell wall self-assembly, and cell elongation. During FU cultivation, the content of L-hydroxyproline in soil increased, especially in CC2 and CC3, and the FU bulbs are also in a rapid growth stage in the second and third years of cultivation. Overall, FU cultivation significantly affected the composition and structure of soil metabolites. This result can be applied to the screening of biomarkers of soil metabolites in rhizosphere soil during FU cultivation.

### 4.3 Correlations between soil metabolism and the microbial community

Effective communication of soil metabolism plays a crucial role in facilitating microorganism-plant interactions (Wang et al., 2019). Furthermore, microbial communities are important drivers of soil metabolism (Li et al., 2020b). Our study found that soil metabolites were more strongly related to bacterial communities than fungal communities (**Figure 7**), although differences in fungal community structure seemed more pronounced during cultivation (**Figures 1**, **2**). We proposed that the metabolites of FU soil had a greater impact on the structure of the fungal community than on that of the bacterial community, while the bacteria played a more significant role in altering the rhizosphere metabolites. Co-occurrence network analysis showed a strong correlations among differentially abundant microorganisms and differentially abundant metabolites, suggesting that microorganisms may interact with metabolites or that metabolites may alter the relative abundance of microorganisms for stress adaptation. Half of the top 10 metabolites with the most significant differences were lipids. They are key compounds of the plasma membrane that promote plant adaptation to abiotic stresses (Wang et al., 2022). Our results demonstrated that these lipids had more negative correlations with differentially abundant microbes (**Figure 8**). Fatty acid biosynthesis and unsaturated fatty acid biosynthesis were significantly enriched among the KEGG pathway. These results implied that lipids affected the composition of microbes during the cultivation of FU. Sucrose was negatively correlated with all the related differentially abundant microorganisms except for *Actinomadura*. Most of these differentially abundant microbial genera were detrimental to plant growth. This implied that a low abundance of sucrose may favor the growth of these pathogenic microorganisms. In addition, the relative abundance of sucrose was positively correlated with that of the beneficial bacterial genus *Actinomadura*, and a low abundance of sucrose was not conducive to the growth of the beneficial bacterial genus *Actinomadura*. Although we have speculated on the relationship between some metabolites and microorganisms, the interactions between microorganisms have not been thoroughly investigated, and more importantly, FU plants were not included in our analysis. In the future, we will combine these results with FU plants to provide better references for improving soil quality and for high-quality cultivation of FU.

## 5 Conclusion

In this study, metagenomics and metabolomics were integrated to reveal FU rhizosphere soil microorganisms and metabolites. The community structure of rhizosphere soil microorganisms was significantly altered during the three-year cultivation of FU, with a decreasing trend in bacterial community diversity and an increasing trend in fungal community diversity. Moreover, the relative abundances of beneficial microorganisms decreased, while the relative abundances of pathogenic microorganisms increased. Soil metabolome expression and metabolic processes were also significantly altered, with the most significant changes in some lipids and sucrose. Microbial community structure was also closely related to the distribution of metabolites in the soil. This study enhances our understanding of the ecological role of metabolites and microbes in FU cultivation.

## Data availability statement

The original contributions presented in the study are publicly available. This data can be found here: https://www.ncbi.nlm.nih.gov/bioproject/PRJNA967213.

## Author contributions

CL and ZL: experimental design. ZL and SC: Fund and resource acquisition. CL: Partial data processing and mapping for initial draft writing. JYu: Provide suggestions and modifications for article writing. JYi and KZ: Provide assistance in some data processing and drawing. ZH supervises this work and provides guidance for it. All authors contributed to the article and approved the submitted version.

## Supplementary material

Supplementary Figure 1 Cluster heat map of the distribution of content between different groups for the top 30 bacterial genera in terms of relative content.

Supplementary Figure 2 Bray-Curtis-based UPGMA clustering maps for bacteria **(A)** and fungi **(B)**.

Supplementary Figure 3 LDA score map obtained from LEfSe analysis: bacterial community **(A)**, fungal community **(B)**.

Supplementary Figure 4 Cluster heat map of content distribution among different groups for the top 30 fungal genera in terms of relative content.

Supplementary Figure 5 Venn diagram of the number of KEGG level 3 pathways among different groups.

Supplementary Figure 6 The top 20 functional compounds with the highest relative content.

Supplementary Figure 7 Correlation heatmap between the top 10 differential genera of bacterial communities and the top 10 differential genera of fungal communities.

Supplementary Figure 8 Heat map of the correlation between microbial genera and the top 30 differential metabolites. bacteria **(A)** and fungi **(B)**.
