# Supplementary figures and images for: Integrated metagenomics and metabolomics analysis reveals changes in the microbiome and metabolites in the rhizosphere soil of Fritillaria unibracteata
Source: Front Plant Sci. 2023 Aug 4;14:1223720. doi: 10.3389/fpls.2023.1223720 (PMC10436506; doi:10.3389/fpls.2023.1223720)

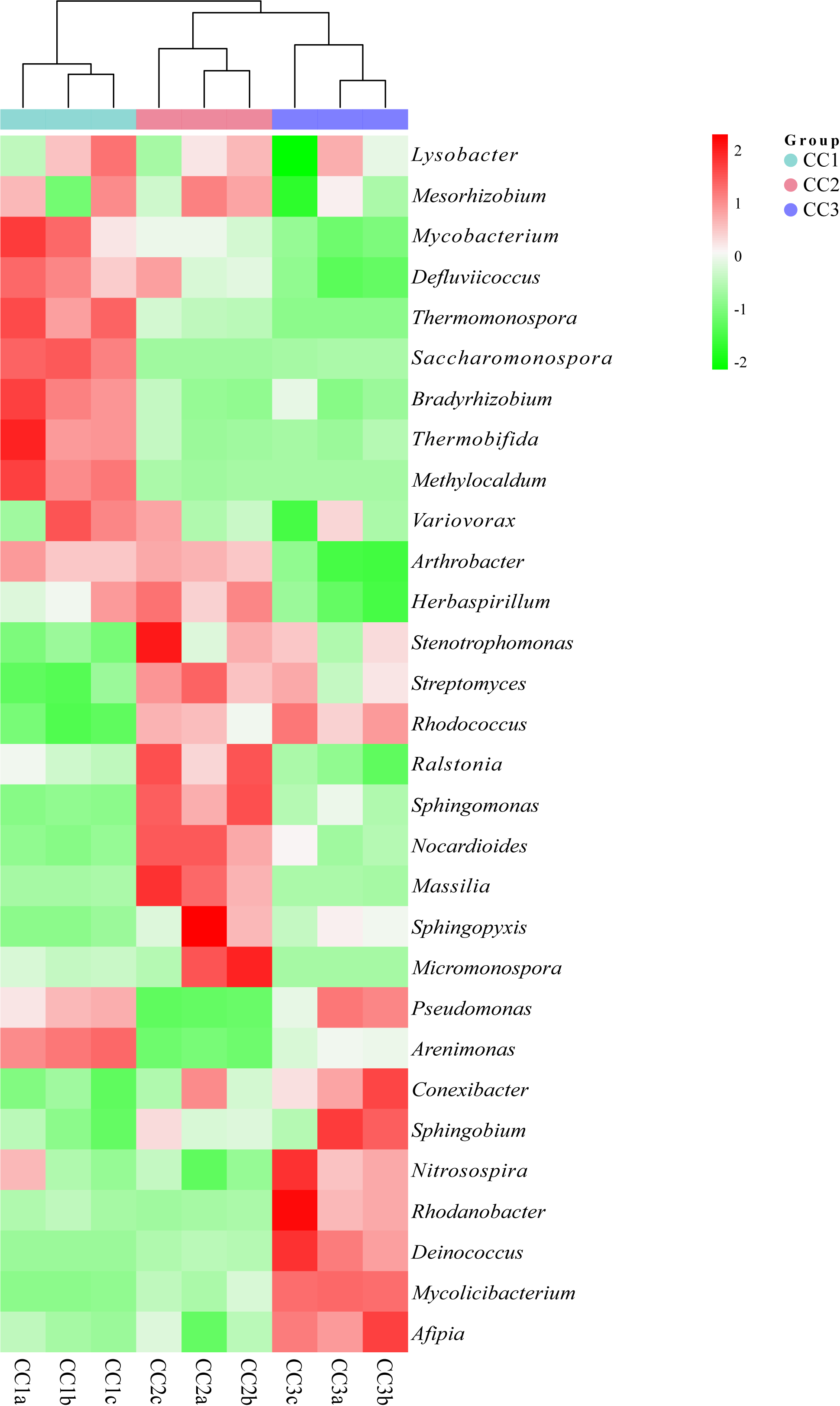

Supplement: Supplementary Figure 1 — Cluster heat map of the distribution of content between different groups for the top 30 bacterial genera in terms of relative content. [file DataSheet_1.zip › Supplementary Figure 1.TIF]

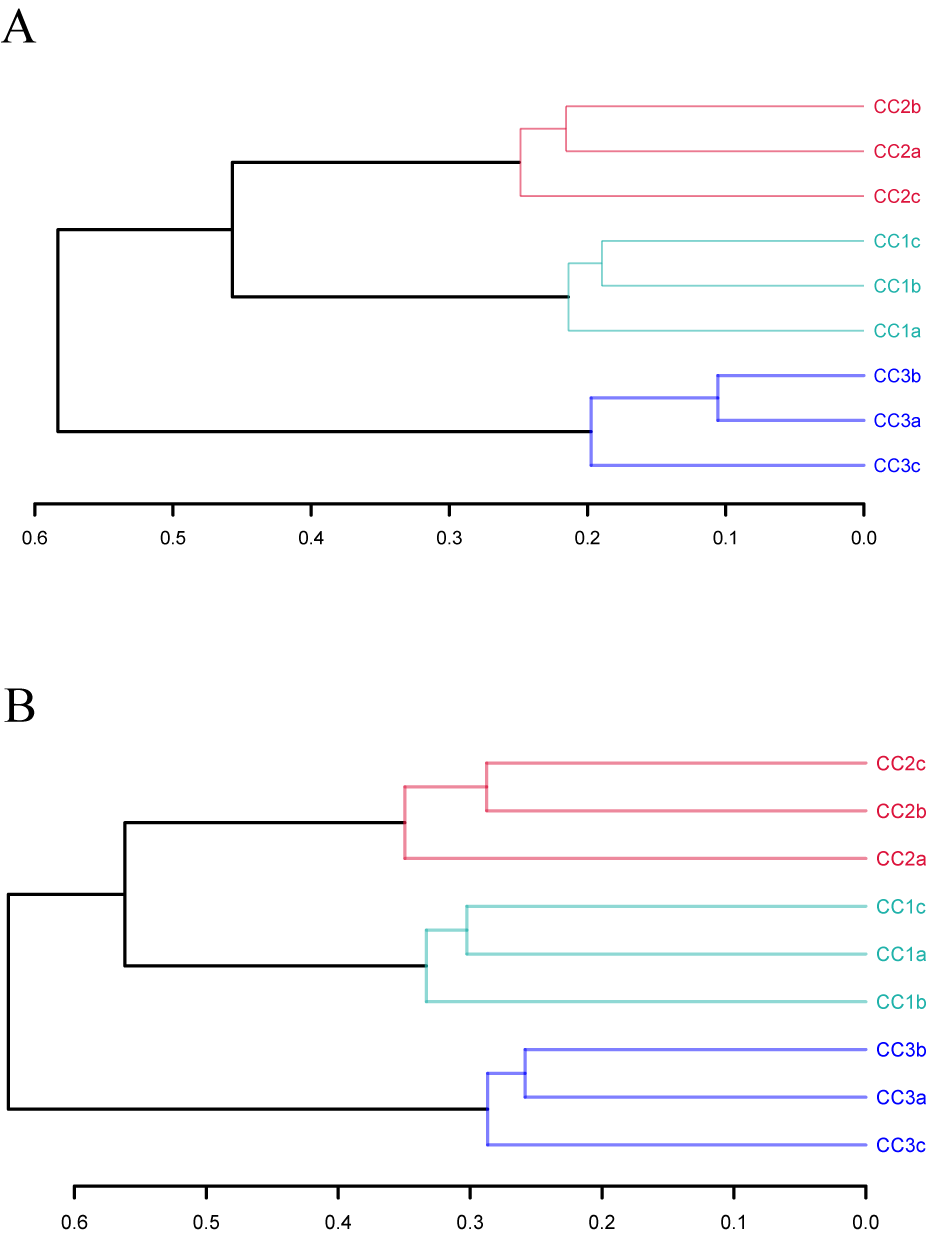

Supplement: Supplementary Figure 1 — Cluster heat map of the distribution of content between different groups for the top 30 bacterial genera in terms of relative content. [file DataSheet_1.zip › Supplementary Figure 2.TIF]

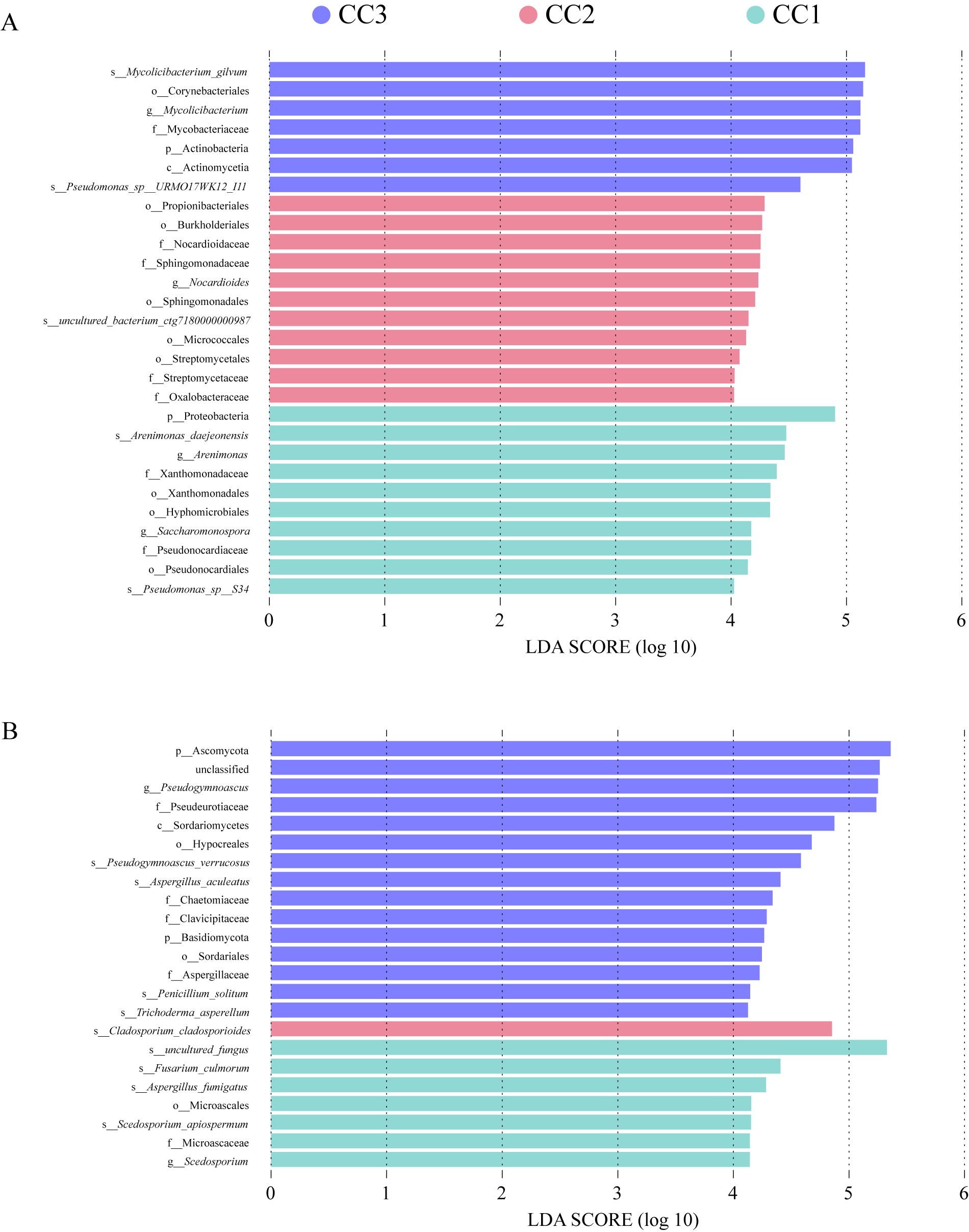

Supplement: Supplementary Figure 1 — Cluster heat map of the distribution of content between different groups for the top 30 bacterial genera in terms of relative content. [file DataSheet_1.zip › Supplementary Figure 3.TIF]

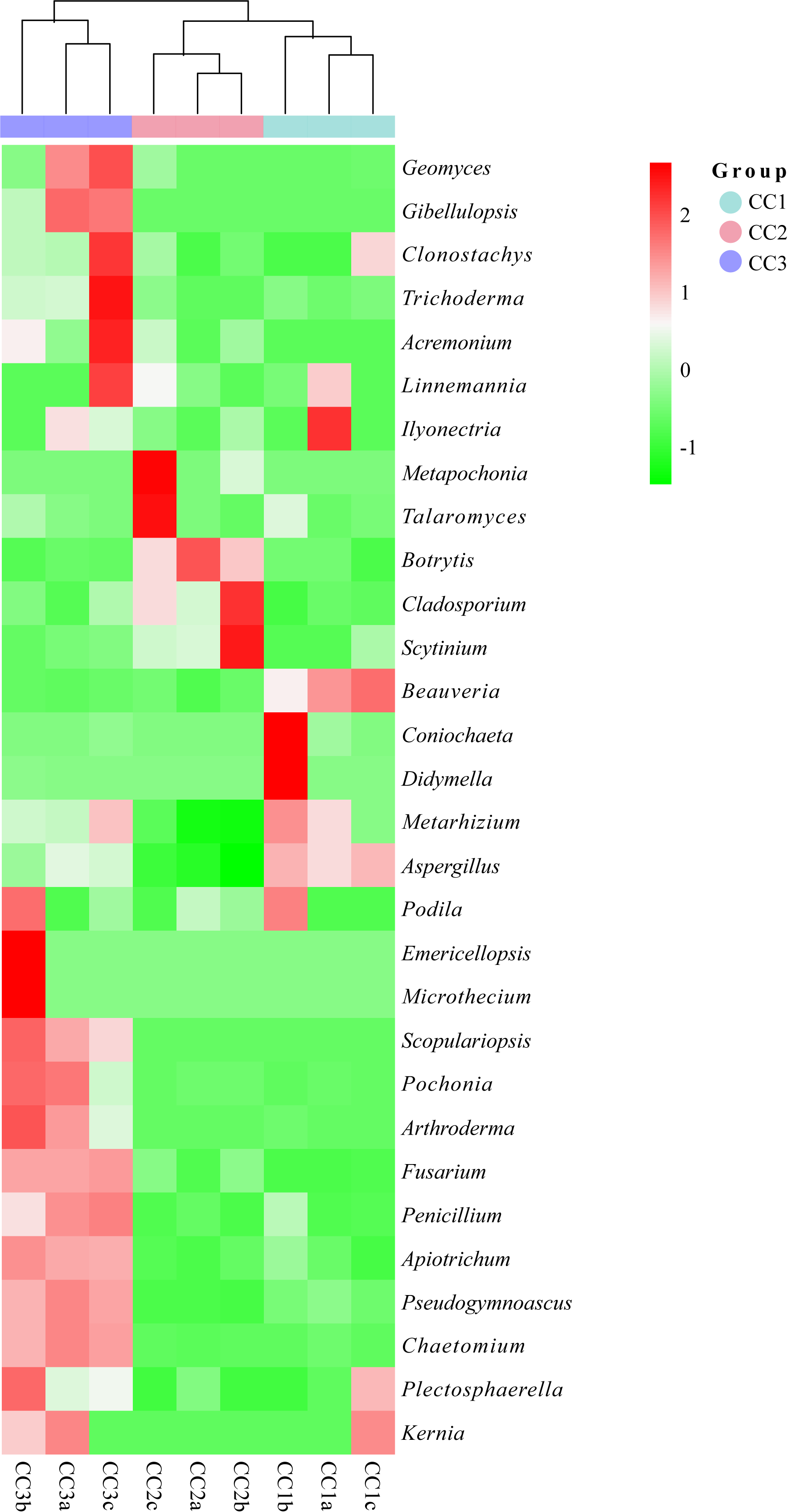

Supplement: Supplementary Figure 1 — Cluster heat map of the distribution of content between different groups for the top 30 bacterial genera in terms of relative content. [file DataSheet_1.zip › Supplementary Figure 4.TIF]

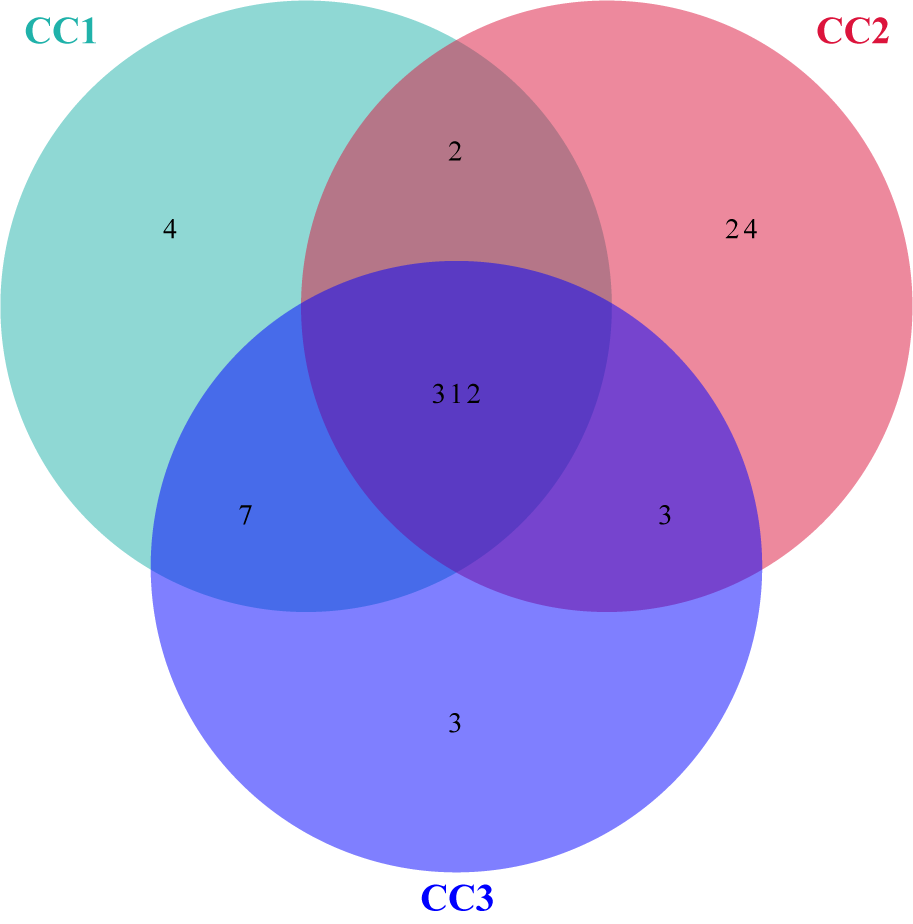

Supplement: Supplementary Figure 1 — Cluster heat map of the distribution of content between different groups for the top 30 bacterial genera in terms of relative content. [file DataSheet_1.zip › Supplementary Figure 5.TIF]

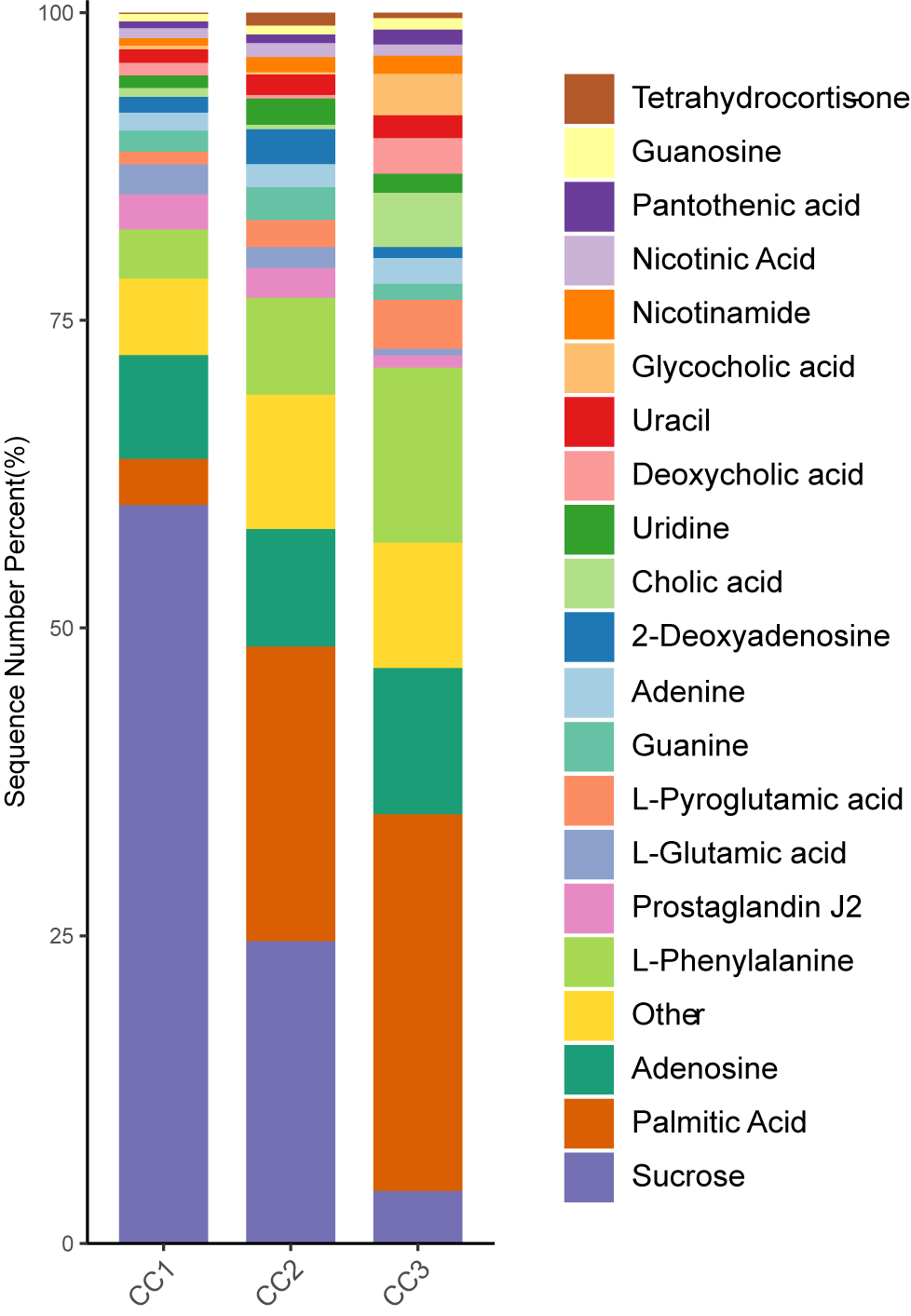

Supplement: Supplementary Figure 1 — Cluster heat map of the distribution of content between different groups for the top 30 bacterial genera in terms of relative content. [file DataSheet_1.zip › Supplementary Figure 6.TIF]

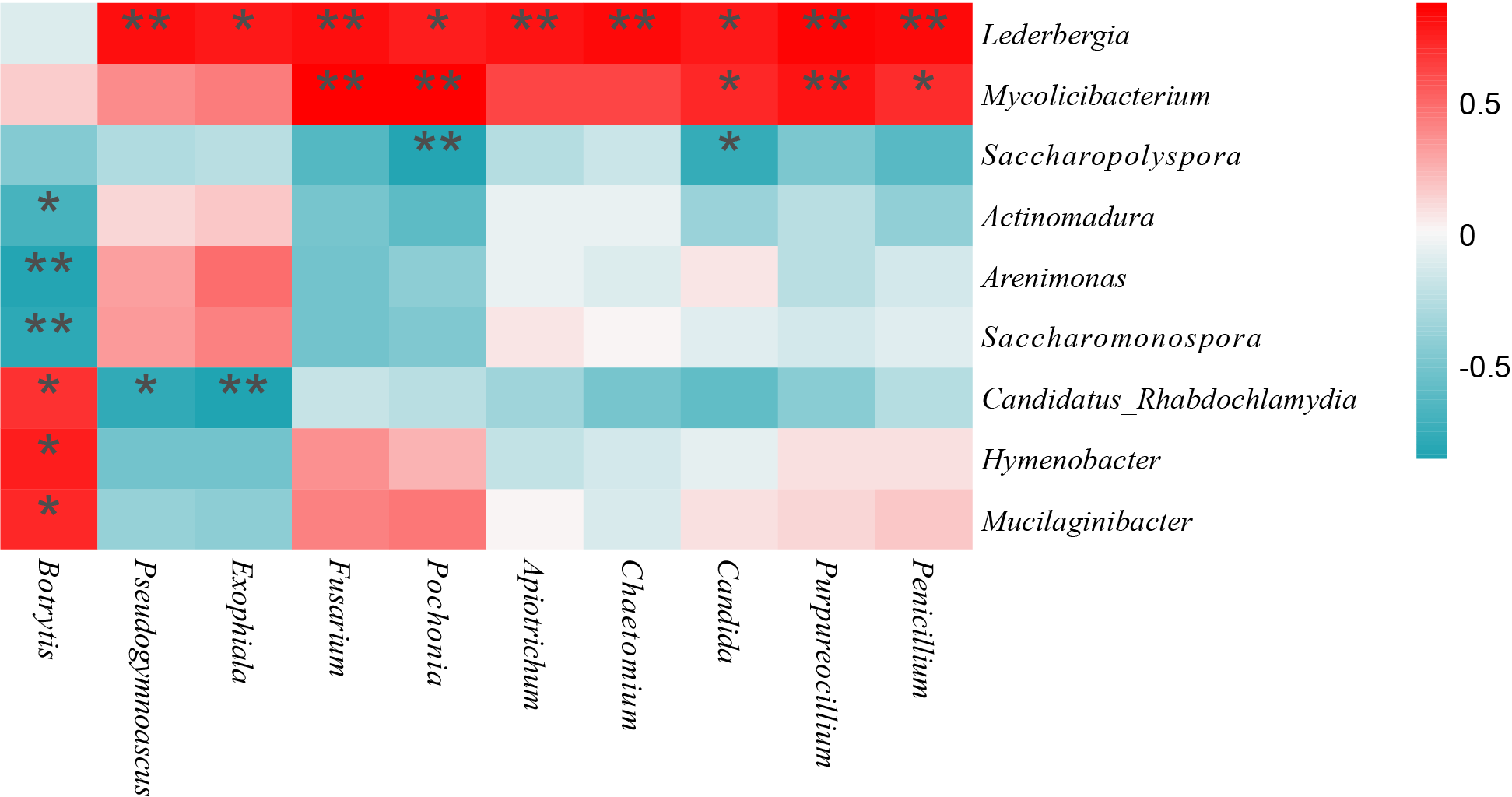

Supplement: Supplementary Figure 1 — Cluster heat map of the distribution of content between different groups for the top 30 bacterial genera in terms of relative content. [file DataSheet_1.zip › Supplementary Figure 7.TIF]

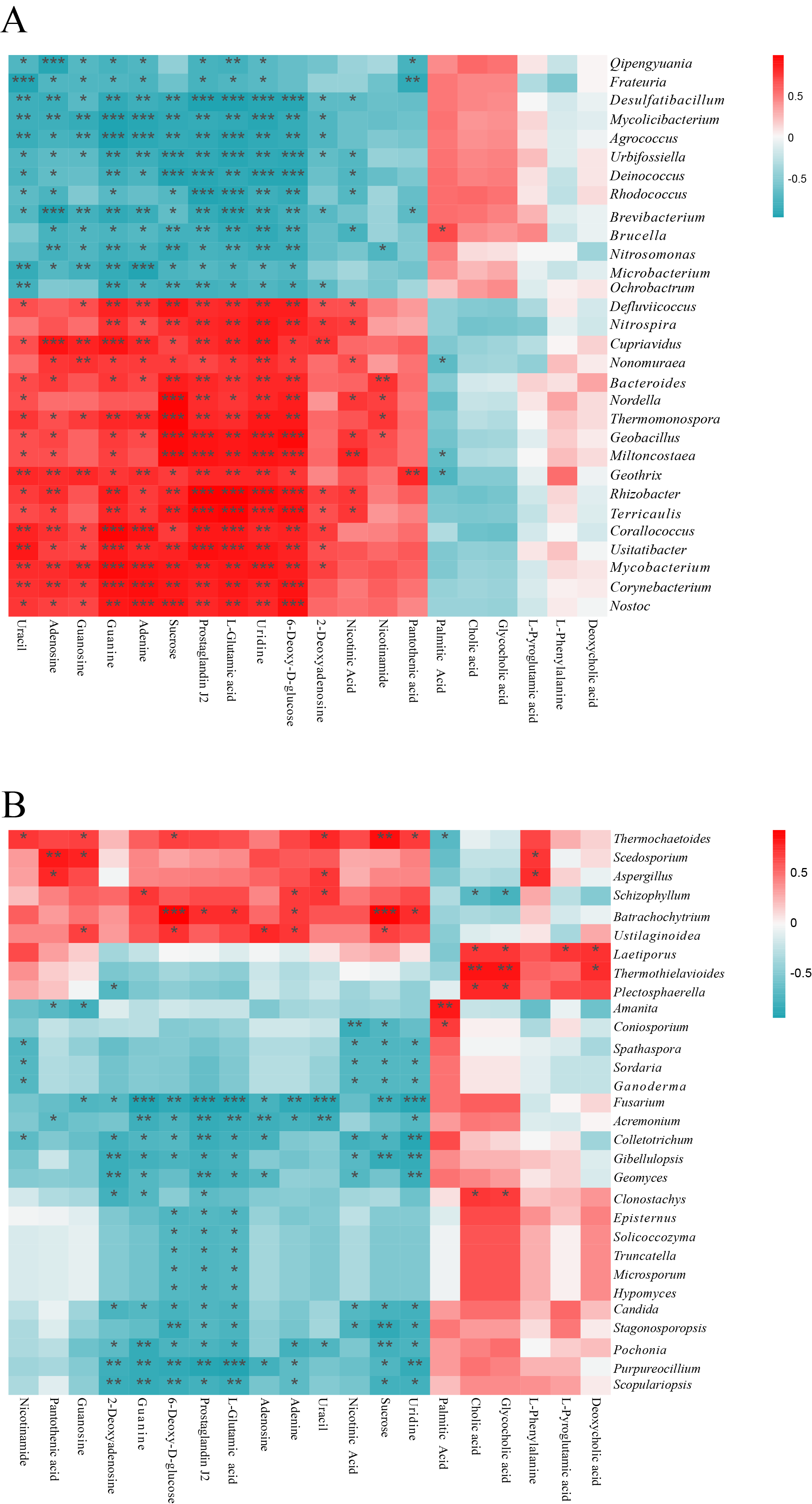

Supplement: Supplementary Figure 1 — Cluster heat map of the distribution of content between different groups for the top 30 bacterial genera in terms of relative content. [file DataSheet_1.zip › Supplementary Figure 8.TIF]
